# Biochemical Characterization of a Novel Exo-Type PL7 Alginate Lyase VsAly7D from Marine *Vibrio* sp. QY108

**DOI:** 10.3390/ijms22168402

**Published:** 2021-08-05

**Authors:** Fengchao Zhang, Zheng Fu, Luyao Tang, Zhelun Zhang, Feng Han, Wengong Yu

**Affiliations:** 1 School of Medicine and Pharmacy, Ocean University of China, Qingdao 266003, China; zhangfengchao@stu.ouc.edu.cn (F.Z.); fuzheng@stu.ouc.edu.cn (Z.F.); tangluyao@stu.ouc.edu.cn (L.T.); zzl9748@stu.ouc.edu.cn (Z.Z.); 2 Laboratory for Marine Drugs and Bioproducts of Qingdao National Laboratory for Marine Science and Technology, Qingdao 266237, China; 3 Key Laboratory of Marine Drugs, Ministry of Education, Qingdao 266003, China; 4 Shandong Provincial Key Laboratory of Glycoscience and Glycotechnology, Qingdao 266003, China

**Keywords:** alginate, alginate lyase, exo-type, monosaccharide, biofuel, brown algae

## Abstract

Brown algae is a kind of renewable resource for biofuels production. As the major component of carbohydrate in the cell walls of brown algae, alginate can be degraded into unsaturated monosaccharide by exo-type alginate lyases, then converted into 4-deoxy-L-*erythro*-5-hexoseulose uronate (DEH) by a non-enzyme reaction, which is an important raw material for the preparation of bioethanol. In our research, a novel exo-type alginate lyase, VsAly7D, belonging to the PL7 family was isolated from marine bacterium *Vibrio* sp. QY108 and recombinantly expressed in *Escherichia coli*. The purified VsAly7D demonstrated the highest activity at 35 °C, whereas it still maintained 46.5% and 83.1% of its initial activity at 20 °C and 30 °C, respectively. In addition, VsAly7D exhibited the maximum activity under alkaline conditions (pH 8.0), with the simultaneously remaining stability between pH 8.0 and 10.0. Compared with other reported exo-type enzymes, VsAly7D could efficiently degrade alginate, poly-β-D-mannuronate (polyM) and poly-α-L-guluronate (polyG) with highest specific activities (663.0 U/mg, 913.6 U/mg and 894.4 U/mg, respectively). These results showed that recombinant VsAly7D is a suitable tool enzyme for unsaturated alginate monosaccharide preparation and holds great promise for producing bioethanol from brown algae.

## 1. Introduction

Currently, due to the deepening problem of resource depletion, macroalgae and microalgae are drawing more attention from researchers owing to their excellent properties as renewable and environmentally friendly feedstocks to produce biofuels [1,2]. Different from terrestrial crops, macroalgae grow in sea water, avoiding the consumption of fresh water, arable soils or fertilizers, and can mitigate atmospheric CO_2_ [3]. Therefore, the exploration of macroalgae and microalgae can relieve the conflict between food and fuels and provide many byproducts, such as edible proteins for poultry [4]. Brown algae, as a species of macroalgae, exists extensively in the ocean. Alginate is the major polysaccharide composition of marine brown macroalgae, accounting for about 40% of the dry weight of the biomass [5]. Based on the differences of the monomers, alginate is divided into poly-α-L-guluronate (polyG), poly-β-D-mannuronate (polyM) and a heteropolymer (polyMG) [6]. Alginate is wildly applied in many industries of food, medicine and cosmetics on the strength of its favorable properties of thickening, gelling and biosecurity [7,8].

Alginate lyases can degrade the 1,4-glycoside bonds of alginate through β-elimination reactions for forming unsaturated double bonds between C4 and C5 at the nonreducing ends. The unsaturated uronic acid residues, existing at the one end of product, are defined as the Δ units [5,9,10,11]. Compared with traditional methods of preparing alginate oligosaccharides (AOS) or alginate monosaccharide under harsh prepared conditions (i.e., long-lasting heating, high pressure and strong acid or base hydrolysis process), enzymatic preparation is considered a mild, efficient, energy-saving and environmentally friendly method [12]. Thus, alginate lyase is an efficient tool to produce alginate oligosaccharides and monosaccharide.

At present, a series of alginate lyases were screened from algae, marine and terrestrial bacteria: microbes from the guts of humans and mollusks [13,14]. Based on the amino acid sequence differences, polysaccharide lyase (PL) was classified into the families of PL5, PL6, PL7, PL8, PL14, PL15, PL17, PL18, PL31, PL32, PL34, PL36, PL39 and PL41 in the Carbohydrate-Active enZYmes (CAZy) database. On the basis of the substrate specificities, alginate lyases can be divided into polyG-specific lyases, polyM-specific lyases and bifunctional lyases [10,11,15]. Furthermore, alginate lyases also possess different action modes, including endo-types or exo-types. Most characterized alginate lyases are endo-type enzymes, which generate unsaturated oligosaccharides of different degrees of polymerizations (≥UDP2). The catalytic efficiencies of endo-type alginate lyases are usually superior to exo-type enzymes [16,17,18]. Exo-type alginate lyases can produce unsaturated monosaccharides, which easily transform into DEH through nonenzymatic reactions [19,20,21]. DEH can be utilized as an energy source by bioengineered *Saccharomyces cerevisiae* and *Escherichia coli* to produce ethanol [22,23,24,25,26]. Therefore, the generation of unsaturated alginate monosaccharide by exo-type alginate lyases is very critical for biofuels production. While the relatively low activities of reported exo-type alginate lyases limit the commercial development of enzymatic biofuel production by using massive brown algae. Thus, it is critical to develop an innovative enzyme that possesses an exo-type mode of action of degrading alginate with high enzymatic activities, simultaneously demonstrating bifunctional lyase properties and tolerant harsh industrial conditions for achieving great advantages in the industrial production of AOS or alginate monosaccharides.

In this research, an innovative PL7 alginate lyase, VsAly7D, was isolated from *Vibrio* sp. QY108, expressed in *E. coli* and characterized. VsAly7D was a nonclassical exo-type enzyme with high activity and simultaneously demonstrated unique characteristics, such as cold-adapted, alkali-resistant, bifunctional and high activity. These characteristics suggested that VsAly7D could be a potential tool enzyme for biofuel production.

## 2. Results and Discussion

### 2.1. Isolation and Bioinformatic Analyzing of VsAly7D

**Figure 1:**
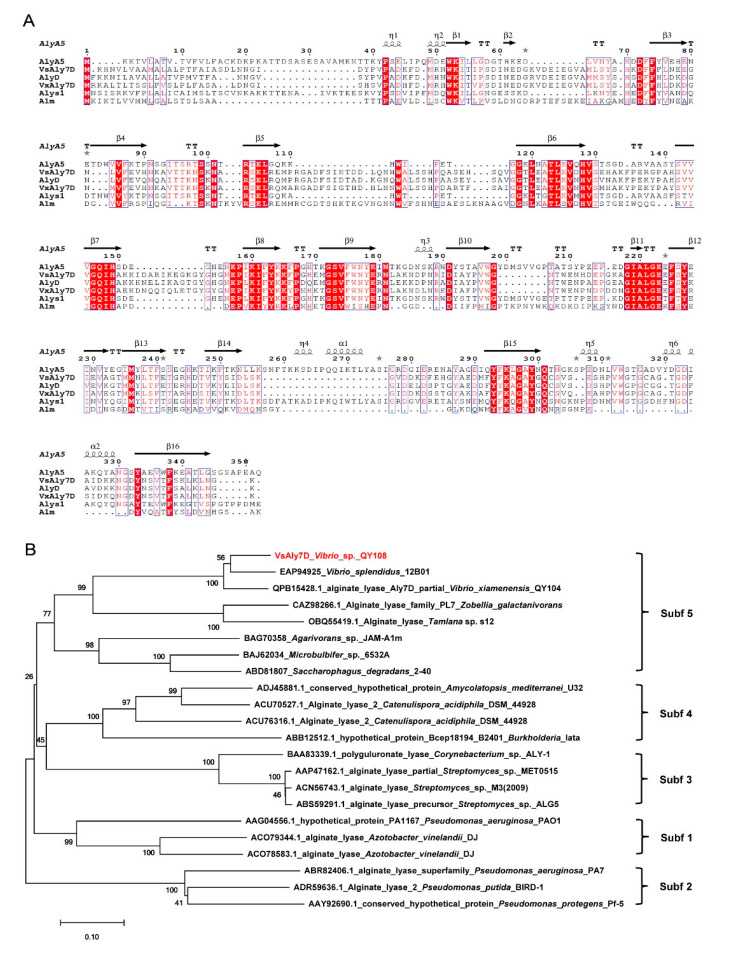
Multiple sequence alignment and phylogenetic analysis of VsAly7D. (**A**) Amino acid alignment of VsAly7D with several identified PL7 alginate lyases, including AlgA5 (CAZ98266) from *Zobellia galactanivorans*, VsAly7D from *Vibrio* sp. QY108 in this study, AlyD (EAP94925) from *Vibrio splendidus* 12B01, VxAly7D (QPB15428) from *Vibrio xiamenensis*, Alys1 (OBQ55419) from *Tamlana* sp. s12 and Alm (BAG70358) from *Agarivorans* sp. JAM-A1m. The asterisk indicate residues with 3D alternate conformations. Red backgrounds represent the same amino acid residues, and red fonts in blue boxes indicate amino acid residues with identity > 70%. (**B**) A phylogenetic analysis of VsAly7D with the other PL7 alginate enzymes.

The alginate lyase gene of *VsAly7D* obtained from marine bacteria *Vibrio* sp. QY108 consisted of an open reading frame (ORF) of 1044 bp and encoded protein VsAly7D, containing 347 amino acid residues. Furthermore, the deduced enzyme protein of VsAly7D had a calculated theoretical Mw of 38,595.07 Da and pI value of 5.65, respectively. VsAly7D contained a signal peptide (Met^1^-Ala^20^) at the N-terminus calculated by SignalP 5.0. Compared with the other reported PL7 alginate lyases, VsAly7D showed the highest identity (73.76%) with VxAly7D (GenBank: QPB15428) from *Vibrio xiamenensis* QY104 [27]. As shown in Figure 1A, VsAly7D contained three highly conserved PL7 regions: R(T/S)EL, QIH and Y(F/Y)K(L/A)G, which contributed to the substrate recognition and catalytic reactions [14,18]. Phylogenetic tree was constructed by the amino acid sequences of VsAly7D and various PL7 alginate lyases (Figure 1B). According to the homology alignment and phylogenetic analysis, the results demonstrated that VsAly7D belongs to the subfamily 5 of PL7.

### 2.2. Homology Modeling and Structure Analysis of VsAly7D

**Figure 2:**
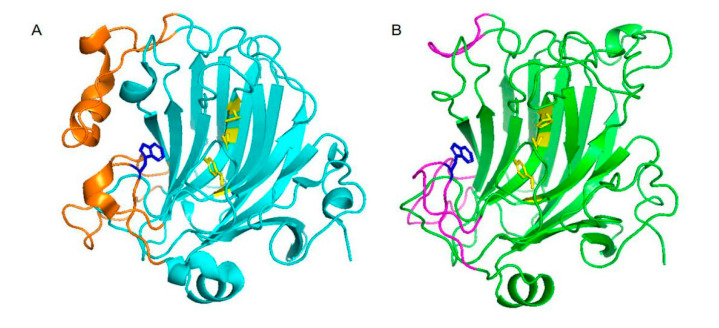
The three-dimensional structure of AlyA5 and VsAly7D. (**A**) The superimposition of the three-dimensional structure of AlyA5. Three loops of AlyA5 (orange cartoon) are highlighted. The catalytic sites of AlyA5 are shown in yellow, and Trp^313^ is shown in blue. (**B**) The superimposition of the three-dimensional structure of VsAly7D. The three loops of VsAly7D (magenta cartoon) are highlighted. The catalytic sites of VsAly7D are shown in yellow, and Trp^315^ is shown in blue.

The structure of VsAly7D was obtained by homology modeling employing AlyA5 (PDB code: 4BE3) from *Zobellia galactaniviorans* DsijT as the template. Through comparing the structure of VsAly7D with AlyA5, the exo-type action mode of VsAly7D was explained. AlyA5 from *Z. galactaniviorans* DsijT was the first reported exo-type PL-7 alginate characterized and structured. VsAly7D showed a highly similar structure with AlyA5, which has 16 reverse-parallel β slices constituting the main structure of the enzyme (Figure 2). The three extra loops (Trp^197^-Asp^217^, Ser^257^-Glu^284^ and Gly^304^-Asp^318^) in AlyA5, sealing off the catalytic groove at one end, hinder the substrate from passing through the catalytic groove that assign its exo-type action mode (Figure 2A). In addition, Trp^313^ is the significant hydrophobic block surrounding the catalytic sites that hold back the extension of hydrophilic groove. Similar to AlyA5, three loops (Trp^227^-Ala^240^, Gly^280^-Glu^287^ and Val^306^-Ala^319^) at the corresponding positions of VsAly7D could be the reason for the exo-type action mode of VsAly7D (Figure 2B). While the loops in the same location of AlyD, which is closest in the phylogenetic analysis, were not prominent, that made them become endo-type enzymes [17]. VsAly7D possesses Trp^315^, which hinders the extension of the hydrophilic groove, like that in AlyA5.

### 2.3. Recombinant Expression and Purification of VsAly7D

**Figure 3:**
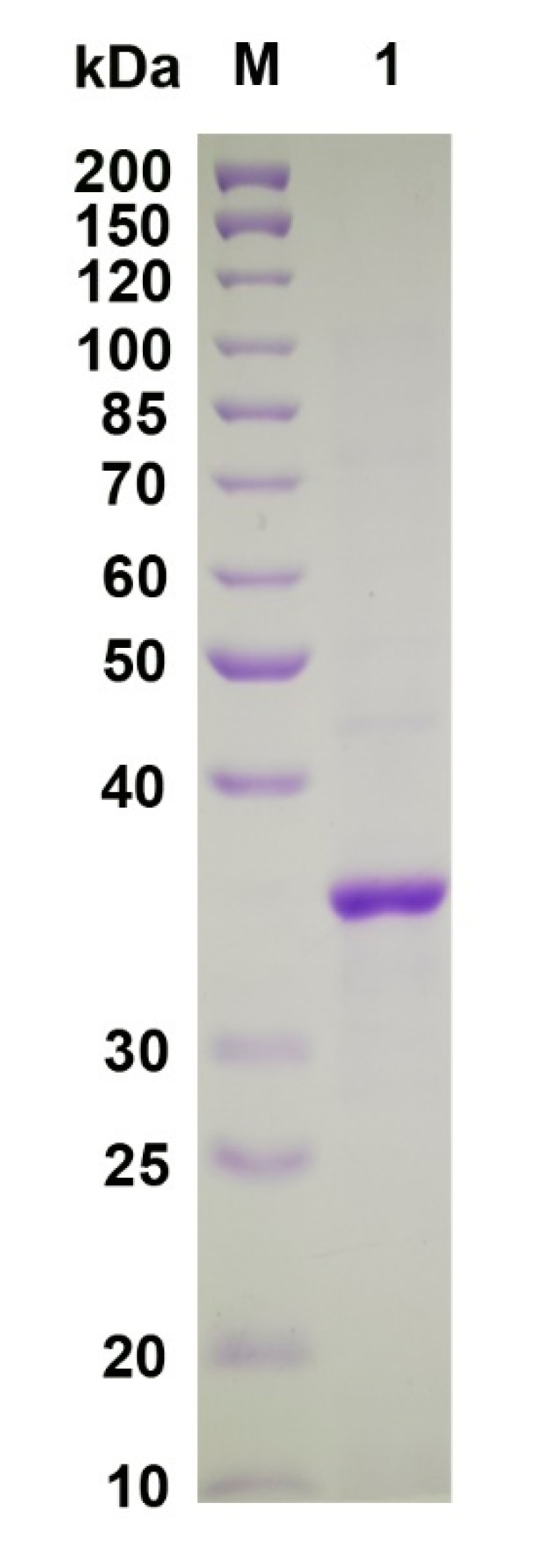
SDS-PAGE of VsAly7D. Lane M, protein standard marker and lane 1, purified VsAly7D.

The *VsAly7D* gene was overexpressed using the pET24a (+)/*E. coli* BL21(DE3) system. Most of the target enzymes were induced and expressed in a soluble form with a high yield (approximately 30,875 U in 1 L of LB medium) after long-term cultivation (24 h) of the recombinant *E. coli* under a low temperature (18 °C). Additionally, VsAly7D was purified to homogeneity with a yield of 78.2% by Ni-affinity chromatography. The SDS-PAGE result displayed a single main band with a protein molecular weight of 37 kDa (Figure 3), catering to the theoretical molecular weight.

### 2.4. Biochemical Characterization of VsAly7D

**Figure 4:**
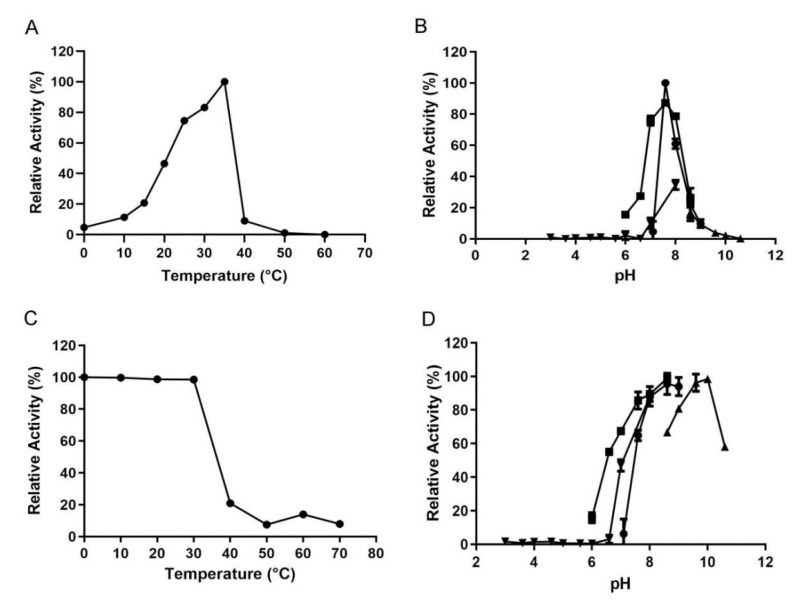
(**A**) The effects of temperatures on VsAly7D activities. (**B**) The optimal pH of VsAly7D in 20-mM buffer (inverted triangle, square, circle and regular triangle represent a citric acid–Na_2_HPO_4_ buffer, Na_2_HPO_4_–NaH_2_PO_4_ buffer, Tris–HCl buffer and Gly–NaOH buffer, respectively). (**C**) The thermostability of VsAly7D. (**D**) The pH stability of VsAly7D in 20-mM buffer (inverted triangle, square, circle and regular triangle represent a citric acid–Na_2_HPO_4_ buffer, Na_2_HPO_4_–NaH_2_PO_4_ buffer, Tris–HCl buffer and Gly–NaOH buffer, respectively).

The optimal temperature of VsAly7D was 35 °C, while it still displayed 46.5% and 83.1% of its initial catalytic activities at 20 °C and 30 °C, respectively (Figure 4A). The activity of recombinant VsAly7D decreased sharply at 40 °C (Figure 4A). In addition, the enzymatic activity was measured after incubating at a series of temperatures for 1 h for assessing the thermal stability of the recombinant VsAly7D. Gratifyingly, the catalytic activity of the alginate lyase still retained 98.7% and 98.6% of the initial activity after incubating at 20 °C and 30 °C, implying that VsAly7D is a cold-adapted enzyme. In contrast, the residual activities of VsAly7D only remained at 21% after storage at 40 °C and barely detectable at temperatures greater than 50 °C (Figure 4C). The reaction temperature is critical to the preparation of alginate oligosaccharides or monosaccharides. Most alginate lyases possess relatively high optimum temperatures exceeding 40 °C [28,29], while cold-adapted enzymes usually have optimal temperature below 35 °C and exhibit high activity below 30 °C [5,30,31], catering to the expectation of energy saving in industrial production. Owing to the excellent biochemical characteristics, we usually give priority to cold-adapted enzymes to produce alginate oligosaccharides or monosaccharides. VsAly7D is a cold-adapted enzyme with a high activity and stability at low temperatures. Heating can quickly terminate the reaction, which is conducive to the concept of energy saving.

The optimal pH of recombinant VsAly7D was pH 7.6 in 20-mM Tris-HCl buffer, and VsAly7D showed similar activity at pH 7.6 in 20-mM Na_2_HPO_4_–NaH_2_PO_4_ buffer (Figure 4B). VsAly7D was stable at pH ranging from 7.6 to 10.6 in different buffers (Figure 4D). Most of the reported alginate lyases exhibited an optimal pH and stability at a neutral environment in the previous study [32], and some alginate lyases—for example, Aly08 from *Vibrio* sp. SY01—have been reported to exhibit high activity and stability under alkaline conditions [33]. VsAly7D was stable even at pH 9.0–10.0 over 12 h, with 80% residual activity. Thus, VsAly7D was an alkaline-stable alginate lyase and more suitable for storage in weak alkaline conditions, which adapts well to harsh environments.

**Figure 5:**
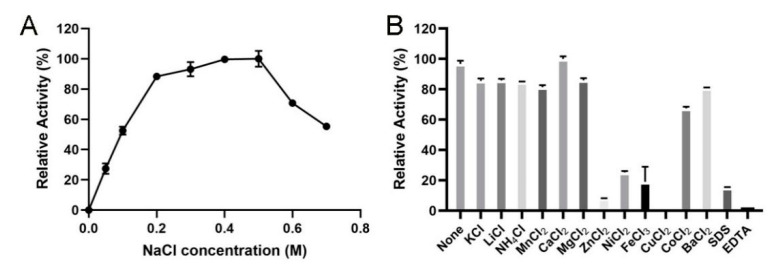
(**A**) The activities of VsAly7D with different the concentrations of NaCl. (**B**) The influences of metal ions, SDS or EDTA on VsAly7D.

The influence of NaCl toward the enzymatic activities of VsAly7D was exhibited in Figure 5A. VsAly7D exhibited high activity in presence of 0.2–0.5-M NaCl, whereas the highest activity was observed following treatment with a high concentration (0.5 M) of NaCl. However, no enzymatic activity of VsAly7D remained without NaCl, indicating that NaCl acted as the strong activator for VsAly7D. The salt-activated property of VsAly7D is conductive to protect the enzyme against harsh high-salt marine environments. Therefore, the substrates, such as brown algae, could be directly decomposed using VsAly7D without the need for desalination. The effect of metal ions, detergents or chelators on the activity of VsAly7D is presented in Figure 5B. Zn^2+^, Fe^3+^, Cu^2+^, SDS and EDTA significantly decreased the activity of VsAly7D (less than 20%) (Figure 5B).

The substrate specificity of VsAly7D was tested using three kinds of polysaccharides. VsAly7D exhibited high activity towards alginate and alginate-derived blocks (polyM and polyG). However, no activity towards other polysaccharides, such as hyaluronan or chondroitin sulfate A, B and C was detected. The specific activities of VsAly7D towards alginate, polyM and polyG were 663.0 U/mg, 913.6 U/mg and 894.4 U/mg, respectively, suggesting that VsAly7D was a bifunctional alginate lyase. VsAly7D exhibited excellent activity that was higher than some typical endo-type PL-7 alginate enzymes, such as AlyA1 and A1m [34,35]. Compared with another excellent exo-type, PL-7, reported in recent research, VsAly7D has better activity than VxAly7D and AlyA5 [27,36]. Although Alys1 exhibited a reported activity of 1350 U/mg, which was higher than that of VsAly7D, VsAly7D showed more excellent temperature and pH stability than Alys1 [37], indicating that VsAly7D is more capable of long-term use. Furthermore, a nonlinear regression analysis was performed to determine the kinetic parameters of VsAly7D. The *K*_m_ and *k*_cat_/*K*_m_ of VsAly7D towards alginate were 0.217 mM and 227 L·mol^−1^·s^−1^, respectively. Bifunctional alginate lyases can degrade different kinds of alginate, suitable for application in the industry.

### 2.5. Action Pattern and End Products of VsAly7D

**Figure 6:**
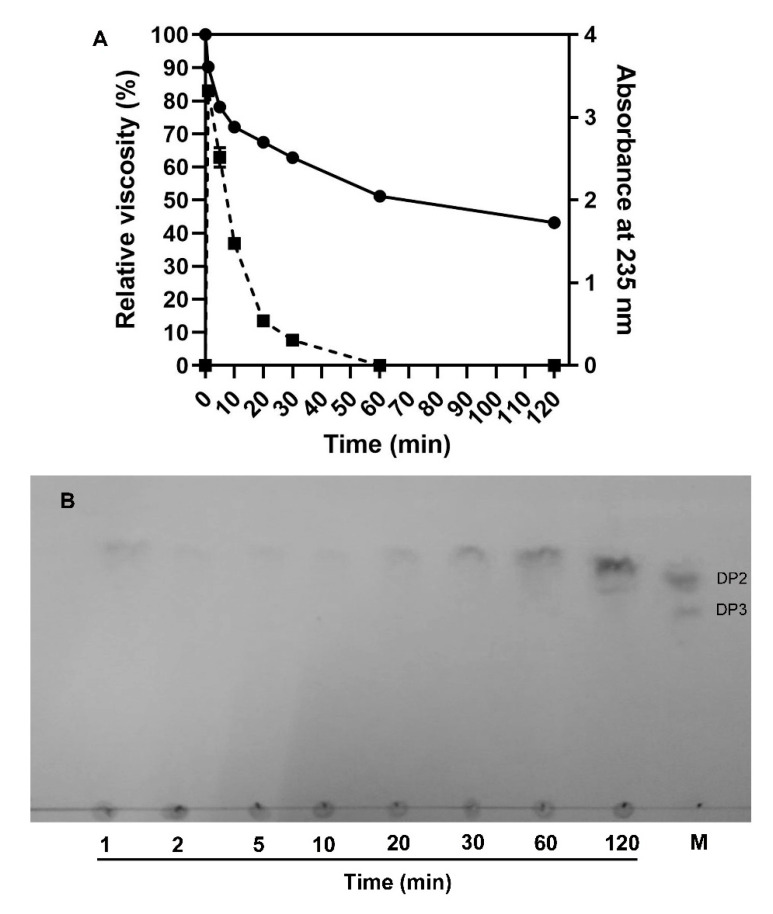
The action pattern of VsAly7D. (**A**) The relative viscosity and the change of A235 during the process of degradation. The solid line represents the relative viscosity, and the dotted line represents A235. (**B**) The time course of the degradation products by VsAly7D analyzed by TLC. M means the maker containing unsaturated alginate disaccharide and trisaccharide.

**Figure 7:**
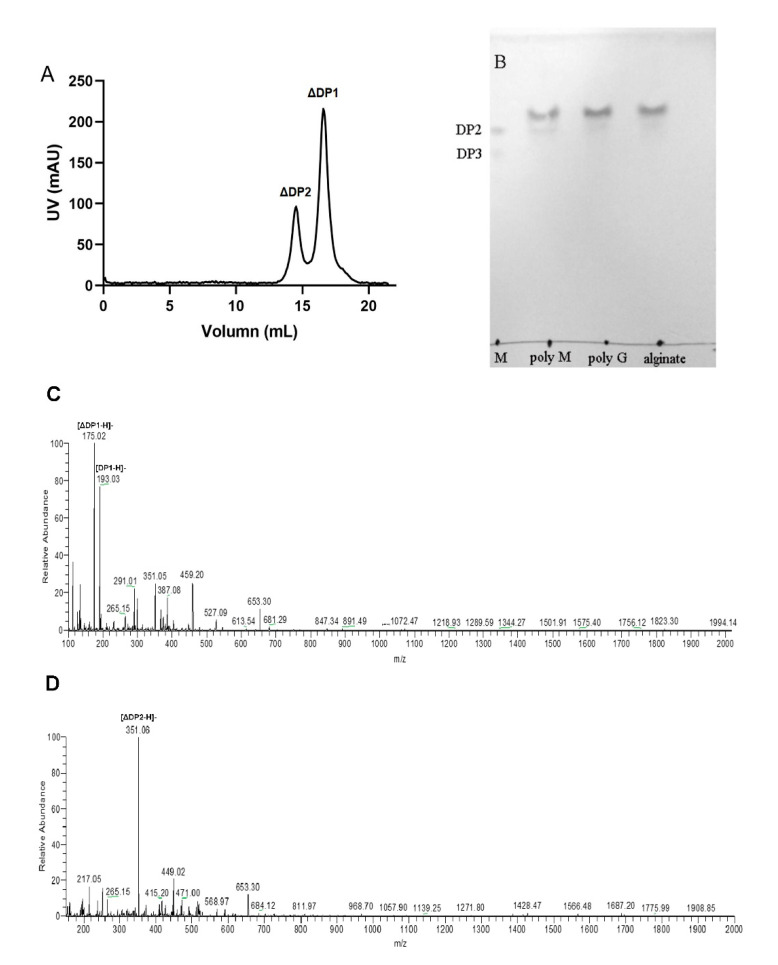
The end product of VsAly7D. (**A**) The end product of alginate evaluated by gel filtration chromatography. (**B**) The end products of polyM, polyG and alginate by VsAly7D degradation. Lane M, unsaturated di- and trisaccharides of the alginate. (**C**) ESI-MS images of the monosaccharide products of the alginate. (**D**) ESI-MS images of the disaccharide products of the alginate.

To explore the action mode of VsAly7D, we measured the change of viscosity and A235 during the process of the reaction (Figure 6A). As time increased, A235 increased rapidly and, subsequently, fell because of unsaturated monosaccharide converted into DEH, which has no absorption at 235 nm. The viscosity declined immediately in the first 5 min and subsequently slowed during the process of the reaction of VsAly7D. At the end of the degradation process (1 h), the viscosity approximately 60% of the initial viscosity remained. The classical endo-type alginate lyases could decrease the viscosity to 10%, whereas the classical exo-type alginate lyases could hardly decrease the viscosity in 1 h. Then, we further used a TLC analysis to investigate the action pattern of VsAly7D. As shown in Figure 6B, VsAly7D only produced unsaturated monosaccharides and a few disaccharides during the degradation process. In addition, the end products of alginate, polyG and polyM degraded by VsAly7D were unsaturated monosaccharides and disaccharides (Figure 7A,B), which were further identified by ESI-MS (Figure 7C,D). These results suggested that VsAly7D exhibited an exo-type action mode.

About twenty bifunctional alginate lyases have been characterized to date, and most of them were endo-type alginates [38]. VsAly7D in this study was an exo-type alginate lyase and presented excellent activity towards the alginate, polyM and polyG, suggesting its potentiality for the preparation of unsaturated monosaccharides.

## 3. Materials and Methods

### 3.1. Materials

Food grade sodium alginates of high viscosity and low viscosity (M/G ratio: 3/5) were bought from Bright Moon Seaweed Group (Qingdao, China). PolyG and polyM (95% purity) were bought from BZ Oligo Biotech Co., Ltd. (Qingdao, China). The Superdex peptide 10/300 GL and Histrap HP purification column (1 mL and 5 mL) were purchased from GE Healthcare (Pittsburgh, PA, USA). Luria-Bertani (LB) culture or Terrific Broth (TB) culture added with kanamycin (30 μg/mL) were used to grow recombinant *E. coli* BL21 (DE3) and DH-5α (TaKaRa, Dalian, China).

### 3.2. Sequence Analysis of Alginate Lyase-Encoding Gene VsAly7D

The signal peptide was calculated by the SignalP 5.0 server (http://www.dtu.dk/services/SignalP/ (accessed on 21 November 2019)). ExPASy (https://web.expasy.org/compute_pi/ (accessed on 21 November 2019)) was used for analyzing the theoretical molecular weight (Mw) and isoelectric point (pI). The conserved domain was investigated by a NCBI CD search (https://www.ncbi.nlm.nih.gov/Structure/cdd/wrpsb.cgi/ (accessed on 21 November 2019)). MEGA 7.0 software was utilized to create a phylogenetic tree through the bootstrapping neighbor-joining method. A multiple sequence alignment was constructed by CLC Genomics Workbench 3 and further aligned by ESPript 3.0 (http://espript.ibcp.fr/ESPript/ESPript/ (accessed on 11 July 2021)). Additionally, the modeled structure of VsAly7D was constructed by SWISS MODEL (https://swissmodel.expasy.org/ (accessed on 21 November 2019)) based on the structure template of AlyA5 (PDB ID: 4BE3) from *Zobellia galactanivorans* Dsij^T^.

### 3.3. Cloning, Expression, and Purification of Recombinant VsAly7D

Based on the genomic sequence of *Vibrio* sp. QY108, two primers towards the *Nde* I site (VsAly7D-F: 5′-GGAGATATACATATGAAACACAACGTACTA-3′) and *Xho* I site (VsAly7D-R: 5′-GTGGTGGTGCTCGAGCTTTCCGTTTAGCTT-3′) were designed. The gene fragment was ligated into plasmid pET-24a (+) and further transformed into *E. coli* BL21(DE3). Using LB broth with 30-μg/mL kanamycin was added to the culture recombinant strain with a temperature of 37 °C until the optical density reached about 0.5 at 600 nm. Subsequently, 0.1-mM isopropyl-β-D-thiogalactoside (IPTG) was used for inducing the protein expression, and the medium was incubated at 18 °C and 160 rpm for 24 h. Then, the fermentation liquids were harvested and disrupted by sonication, followed by centrifuged to remove bacteria debris and unbroken bacterial cells for obtaining the crude enzyme. Subsequently, the obtained enzyme was purified through a HisTrap^TM^ HP column (GE Healthcare, Pittsburgh, PA, USA), in which the purity and Mw of VsAly7D were further analyzed by 10% sodium dodecyl sulfate polyacrylamide gel electrophoresis (SDS-PAGE). In addition, the protein concentration was calculated by the BCA protein assay kit (Vazyme Biotech Co., Ltd., Nanjing, China).

### 3.4. Enzymatic Activity Assay

The reaction system was performed by rapidly adding the suitable diluted VsAly7D (100 μL) with 900 μL 0.3% (*w*/*v*) standard sodium alginate substrate (20-mM Na_2_HPO_4_-NaH_2_PO_4_ buffer that contained 300-mM NaCl, pH 7.6) and then conducted at 30 °C for 10 min. The activities were measured by monitoring the change of absorbance at 235 nm (A235) as a Δ-4,5-unsaturated double bond formed during the reaction process. A235 were measured by a double-beam spectrophotometer UH5300 (Hitachi High-Technologies Co., Tokyo, Japan). One unit of alginate lyase activity (U) was the required amount of enzymes causing the augmented A235 value of 0.1 per minute under the aforementioned conditions.

The kinetic parameters of VsAly7D were investigated by using various concentrations of sodium alginate (0.05–7.5 mg/mL). The *K_m_* and *K_cat_* were evaluated by GraphPad Prism 8 by the method of a nonlinear regression analysis.

### 3.5. Biochemical Characterization of VsAly7D

The influence of the pH value toward the enzymatic activities of VsAly7D was performed in different buffers, including Na_2_HPO_4_–citric acid (20 mM, pH 3.0–8.0), Na_2_HPO_4_–NaH_2_PO_4_ (20 mM, pH 6.0–8.0), Tris–HCl (20 mM, pH 7.05–8.95) and 20-mM glycine–NaOH (pH 8.6–10.6). In addition, the pH stability of VsAly7D was measured in different aforementioned buffers after being incubated at 4 °C for 12 h. The reaction system was then performed at different temperatures (10–60 °C) to evaluate the optimal catalytic temperature of VsAly7D. The thermostability of VsAly7D was assessed according to the measurement of residual activities after being incubated with phosphate buffer (20 mM, pH 7.3) at a series of temperatures (0–70 °C) for 1h. The influences of metal ions and chelators on the VsAly7D activities were also investigated by testing the activities of VsAly7D in the reaction system after adding different metals or chelator compounds, with a final concentration of 1 mM.

### 3.6. Analysis of Action Pattern

The thin-layer chromatography (TLC) method was utilized for analyzing the action patterns of VsAly7D. Concretely, 1 mL of purified VsAly7D (10 U) was mixed with 9 mL of 0.3% (*w*/*v*) standard sodium alginate and incubated at 30 °C for 0, 1, 2, 5, 10, 20, 30, 60 or 120 min, respectively. Then, the degradation products of VsAly7D were separated by a TLC plate (Merck, Darmstadt, Germany) by a mixture solution (1-butanol/formic acid/water, 4:6:1, by vol.) and sprayed by a 1,3-naphthalenediol reagent (1-mg 1,3-naphthalenediol dissolved in a buffer of ethanol/water/concentrated sulfuric acid, 5:4:1, by vol.), which then heated to 130 °C for 5 min.

### 3.7. Analysis of End Products

The end products of VsAly7D were investigated by fast protein liquid chromatography (FPLC) and negative-ion electrospray ionization mass spectrometry (ESI-MS). In general, the samples were prepared by incubating 1-mL enzymes (10 U) with 9 mL of 0.3% (*w*/*v*) standard sodium alginate substrate at 30 °C for 12 h. Subsequently, the degradation products were separated by a Superdex peptide 10/300 gel filtration column (GE Healthcare, Pittsburgh, PA, USA) and monitored at A235 supported by the FPLC system, in which 0.2-M ammonium bicarbonate was used as the mobile phase with a 0.2-mL/min flow rate. The whole system was run by the software UNICORN 5.31. The separated products were lyophilized, and the molecular masses of the end products were analyzed by ESI-MS.

## 4. Conclusions

In this study, an innovative exo-type PL-7 alginate lyase, VsAly7D, was cloned and characterized from *Vibrio* sp. QY108. VsAly7D is a PL-7 alginate lyase with a bifunctional exo-type action mode and exhibited the high activities towards the sodium alginate, polyG, and polyM compared to the other reported enzymes that exolytically degraded the alginate. VsAly7D displayed excellent specific activity and stability at room temperature and in an alkaline environment. Therefore, VsAly7D will be a potential enzyme to produce unsaturated alginate monosaccharides, which can spontaneously be converted to DEH, a kind of raw material for biofuel production.

## 5. Patents

There is a Chinese invention patent, ZL202010041545.2, resulting from the work reported in this manuscript.

## Data Availability

Data is contained within the article.
